# Development and validation of a novel risk classification tool for predicting long length of stay in NICU blood transfusion infants

**DOI:** 10.1038/s41598-024-57502-3

**Published:** 2024-03-22

**Authors:** Nurbiya Arkin, Ting Zhao, Yanqing Yang, Le Wang

**Affiliations:** Department of Neonatal, The First Affiliated Hospital of Xinjiang Medical University, Urumqi, 830054 Xinjiang China

**Keywords:** Blood transfusion infants, Long length of stay (LOS), Prediction nomogram, Medical research, Paediatric research

## Abstract

Newborns are as the primary recipients of blood transfusions. There is a possibility of an association between blood transfusion and unfavorable outcomes. Such complications not only imperil the lives of newborns but also cause long hospitalization. Our objective is to explore the predictor variables that may lead to extended hospital stays in neonatal intensive care unit (NICU) patients who have undergone blood transfusions and develop a predictive nomogram. A retrospective review of 539 neonates who underwent blood transfusion was conducted using median and interquartile ranges to describe their length of stay (LOS). Neonates with LOS above the 75th percentile (P75) were categorized as having a long LOS. The Least Absolute Shrinkage and Selection Operator (LASSO) regression method was employed to screen variables and construct a risk model for long LOS. A multiple logistic regression prediction model was then constructed using the selected variables from the LASSO regression model. The significance of the prediction model was evaluated by calculating the area under the ROC curve (AUC) and assessing the confidence interval around the AUC. The calibration curve is used to further validate the model’s calibration and predictability. The model’s clinical effectiveness was assessed through decision curve analysis. To evaluate the generalizability of the model, fivefold cross-validation was employed. Internal validation of the models was performed using bootstrap validation. Among the 539 infants who received blood transfusions, 398 infants (P75) had a length of stay (LOS) within the normal range of 34 days, according to the interquartile range. However, 141 infants (P75) experienced long LOS beyond the normal range. The predictive model included six variables: gestational age (GA) (< 28 weeks), birth weight (BW) (< 1000 g), type of respiratory support, umbilical venous catheter (UVC), sepsis, and resuscitation frequency. The area under the receiver operating characteristic (ROC) curve (AUC) for the training set was 0.851 (95% CI 0.805–0.891), and for the validation set, it was 0.859 (95% CI 0.789–0.920). Fivefold cross-validation indicates that the model has good generalization ability. The calibration curve demonstrated a strong correlation between the predicted risk and the observed actual risk, indicating good consistency. When the intervention threshold was set at 2%, the decision curve analysis indicated that the model had greater clinical utility. The results of our study have led to the development of a novel nomogram that can assist clinicians in predicting the probability of long hospitalization in blood transfused infants with reasonable accuracy. Our findings indicate that GA (< 28 weeks), BW(< 1000 g), type of respiratory support, UVC, sepsis, and resuscitation frequency are associated with a higher likelihood of extended hospital stays among newborns who have received blood transfusions.

## Introduction

Newborns have become the main blood transfusion population due to their small blood volume and underdeveloped hematopoietic system. As the survival rate of newborns continues to improve^1^, there has been a significant increase in the number of infants requiring long-term stays in the neonatal ward. Consequently, over the past few years, the overall transfusion rate has experienced exponential growth^2^. Nearly 58% of low birth weight infants and more than 90% of very low birth weight infants need at least one blood transfusion during hospitalization^3^. Providing red blood cell (RBC) transfusions can increase the capacity of tissues to carry oxygen, thereby reducing the likelihood of apnoeic episodes and promoting weight gain and growth among premature newborns^4^.

Nevertheless, an increasing body of evidence suggests a link between transfusion exposure and negative outcomes. The existing studies indicate that blood transfusion is not only closely related to the mortality of children, but also may be related to the occurrence of premature complications such as intraventricular hemorrhage (IVH), bronchopulmonary dysplasia (BPD), necrotizing enterocolitis (NEC)^5–7^. These complications not only threaten the life of newborns, but also lead to long hospitalization. Some studies showed that transfusion infants have longer hospital or NICU length of stay than non-transfusion group^8,9^. The long hospitalization exposes them to medical environments for a longer duration, thereby increasing the risk of hospital-acquired infections and other complications^10^. Additionally, it places a heavy burden on neonatal care. Moreover, an extended LOS may hinder the establishment of parental bonding and interaction with the newborn, potentially causing significant emotional and financial stress on the family^11^. Therefore, the long LOS of these patients in the neonatal intensive care unit has become a worrisome issue, making accurate prediction of LOS in the neonatal ward increasingly crucial. Furthermore, there is currently limited research on LOS specifically focused on transfusion patients.

Indeed, our objective is to utilize data from the NICU to explore the predictor variables that may lead to extended hospital stays in NICU patients who have undergone blood transfusions and develop a predictive nomogram, in order to provide more evidence for the prevention of long hospital stays and the optimization of resource allocation for NICU patients.

## Methods

This study aims to conduct a retrospective investigation on the data of registered newborns at the First Affiliated Hospital of Xinjiang Medical University. The research protocol received approval from the Ethics Committee of the First Affiliated Hospital of Xinjiang Medical University in Urumqi. Given the retrospective nature of the study, the necessity for a written informed consent form has been waived.

### Study population

Between May 1, 2021, and May 1, 2023, neonates who received blood transfusions at the First Affiliated Hospital of Xinjiang Medical University were included in this study. The following criteria were applied for inclusion in the study: 1. Neonates who received at least one blood transfusion during their hospitalization; 2. Admission age less than 1 day and 3. Admitted to the NICU rather than general wards. The following exclusion criterion was applied in this study: 1. Rapid discharge or non-prescription discharge for non-medical reasons; 2. Neonatal death during NICU hospitalization or before admission; 3. Chromosomal abnormalities or severe congenital malformations; 4. Neonates undergoing surgery and 5. Patients with missing data exceeding 10% in general conditions, complications, and laboratory measurements are excluded.

### Data collection

Data collection from the enrolled neonates encompassed the following 57 variables: (1) General conditions of the neonate—GA (< 28 weeks), BW(< 1000 g), gender, 1-min and 5-min Apgar scores, feeding patterns, respiratory support type (invasive ventilation, noninvasive ventilation, others: nasal cannula oxygen inhalation or anaerobic), antenatal glucocorticoids, umbilical venous catheterization (UVC), amniotic fluid contamination, and placental abruption. (2) Complications—neonatal respiratory distress syndrome (NRDS), NEC, IVH, pneumorrhagia, sepsis, congenital heart disease (CHD), hemolytic jaundice, and rescue frequency (≥ 3 times). (3) Laboratory parameters ()- white blood cell (WBC) count, platelet (PLT) count, red cell distribution width (RDW), hematocrit (HCT), neutrophils, lymphocytes, procalcitonin (PCT), C-reactive protein (CRP), interleukin (IL), potassium (K), sodium (Na), potential of hydrogen (pH), arterial partial pressure of carbon dioxide (PaCO2), arterial partial pressure of oxygen (PaO2), bicarbonate (HCO3), total bilirubin (TBIL), albumin (ALB), aspartate aminotransferase (AST), alanine aminotransferase (ALT), lactate dehydrogenase (LDH), creatine kinase (CK), lactic acid (LA), triglyceride (TG), total cholesterol (TC), high-density lipoprotein (HDL), low-density lipoprotein (LDL), creatinine, and urea. (4) Maternal factors—maternal age, primipara, multiple births, cesarean section, pregnancy anemia, hypertensive disorders of pregnancy (HDP), intrahepatic cholestasis of pregnancy (ICP), gestational diabetes mellitus (GDM), hypothyroidism, and idiopathic thrombocytopenic purpura (ITP). The laboratory data utilized in this study were derived from the initial blood tests conducted in the NICU. We imputed the missing data (< 10%) using the MICE package (version 3.14.0)^12^.

### Definitions

We defined long LOS as exceeding the 75th percentile of LOS^13,14^. The original continuous variables of gestational age and weight did not show significant differences (*p* > 0.05). Therefore, we explored various combinations, including different categories for weight and gestational age classifications. In the end, we found that the classification method using gestational age (< 28 weeks, ≥ 28 weeks) and weight (< 1000 g,  ≥ 1000 g) yielded more significant differences for our study.

### Development and assessment of the nomogram

In this study, the subjects were randomly split into groups, with two-thirds of the subjects assigned to the training set, which was used to identify the predictor variables associated with long hospitalization in newborns who received blood transfusions and to develop a prediction scoring model. The remaining one-third of the subjects were designated as the validation set, which was used to evaluate the effectiveness of the prediction scoring system. This study utilized the least absolute shrinkage and selection operator (LASSO) method to identify the optimal predictive variables among the predictor variables present in newborns who received blood transfusions^15^. LASSO regression, which incorporates L1 regularization, is particularly well-suited for datasets with highly correlated predictor variables. By introducing a penalty term, LASSO regression tends to select a subset of variables while shrinking the coefficients of other highly correlated variables towards zero, effectively mitigating the impact of multicollinearity. Therefore, in our study, we employed LASSO regression to handle the presence of highly correlated predictors and to provide more stable and reliable estimates. The LASSO regression model was employed to select variables that had nonzero coefficients, which were then used in a multivariable logistic regression analysis to construct a predictive model for long LOS. A predicting model nomogram was created by incorporating all the potential predictors selected in the LASSO regression model. Moreover, the diagnostic performance of the visual prediction model was externally validated using the *Hosmer–Lemeshow* test and coefficient of determination (R2) to evaluate its goodness of fit. The model’s predictive accuracy and conformity were also assessed examining the shape of the ROC and calibration curves and by using metrics such as the area under the ROC curve (AUC). Additionally, the decision curve analysis (DCA) was utilized to assess the net benefit of the model for patients. The discrimination and calibration of the model were checked through bootstrapping with 1000 resamples. Finally, to enhance the credibility and accuracy of our model evaluation, we utilized a technique called fivefold cross-validation. This method involves dividing our dataset into five subsets of approximately equal size. During the evaluation process, we iteratively trained our model on four of these subsets while using the remaining subset as a validation set. This allowed us to assess the model’s performance across multiple iterations, each time using a different subset for validation.

### Statistical analysis

Statistical analysis was conducted using R software, Version 4.1.3 (available at https://www.Rproject.org). Categorical data are presented as numbers and percentages, while continuous variables are reported as mean ± standard deviation (SD) if they follow a normal distribution, or as median (interquartile range [IQR]) if they do not. To assess proportions, the χ2 test or Fisher’s exact test was used for comparing categorical variables. For continuous variables that exhibited a normal distribution, independent group t-tests were employed to compare means. All statistical tests were performed in a two-sided manner, and *p*-values ≤ 0.05 were considered statistically significant.

### Ethics approval and consent to participate

This study followed the Helsinki Declaration and was approved by the Ethics Committee board of Xinjiang Medical University Affiliated First Hospital. Due to the retrospective nature of the study, the need of informed consent was waived by the Ethics Committee board of Xinjiang Medical University Affiliated First Hospital. All methods were carried out in accordance with relevant guidelines and regulations. No biological specimens were used in this study.

## Results

### Baseline characteristics of included neonates

**Figure 1:**
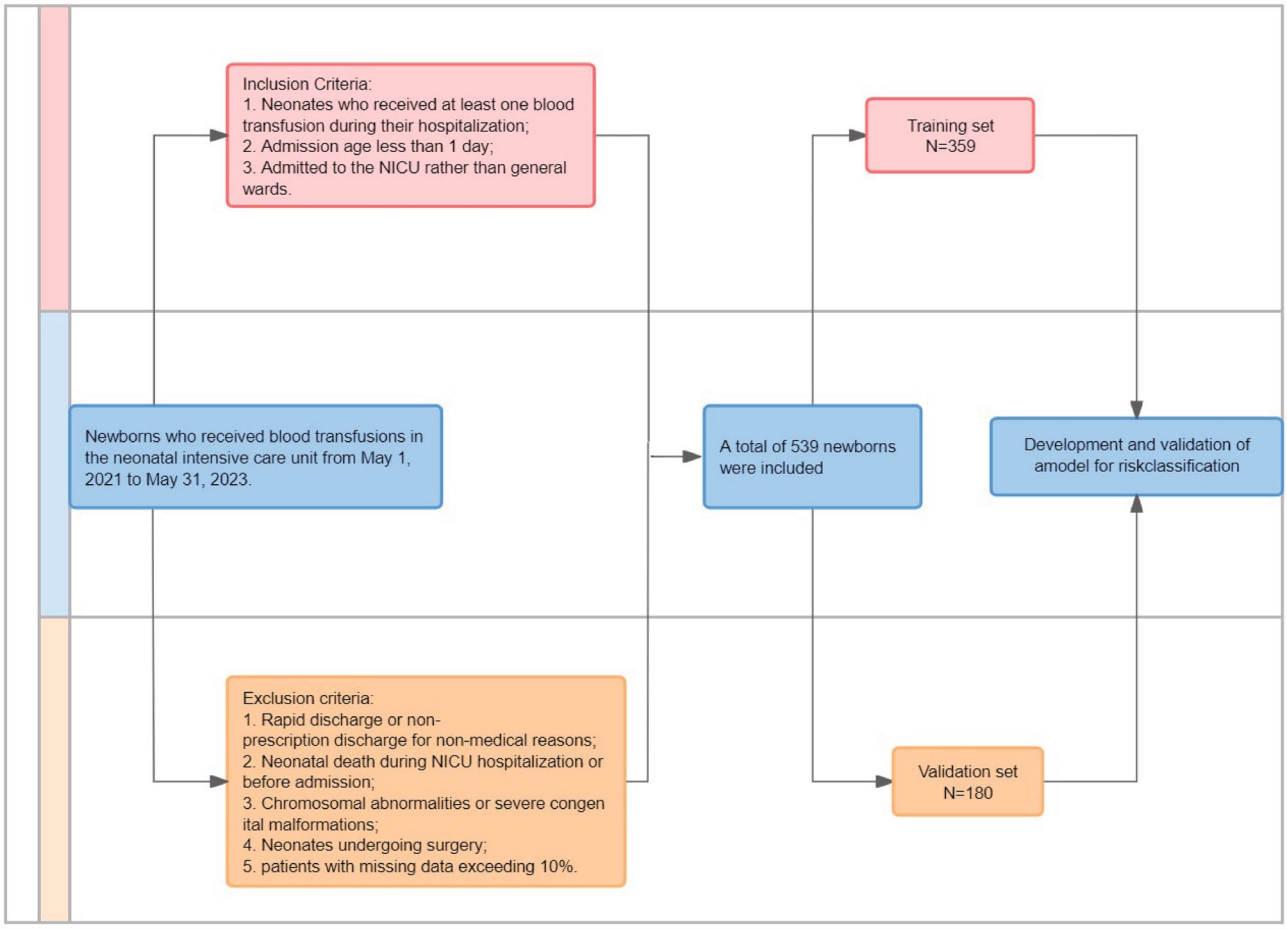
Study flowchart.

The study analyzed a total of 539 infants, among whom 398 had hospital stays shorter than the 75th percentile (normal LOS), while 141 infants had hospital stays longer than the 75th percentile (long LOS). (Fig. 1) The baseline characteristics of these two groups are presented in Table 1. Compared with children in the normal LOS group, a significantly higher proportion of children in the long LOS group had a gestational age of less than 28 weeks (19.9% vs. 1.76%; *p* < 0.001) and weight less than 1000 g (29.8% vs. 2.01%; *p* < 0.001). A statistically significant difference (*p* < 0.05) was observed between the two groups in terms of Apgar score, feeding patterns, respiratory support, UVC, RDS, NEC, pneumorrhagia, sepsis, rescue frequency (≥ 3 times), HCO3, ALB, CK, and urea, as illustrated in Table 1.

**Table 1:** Baseline characteristics of patients.

Variables	All	Normal	Long	*p*-value
	N = 539	N = 398	N = 141	
GA (weeks)				< 0.001
≥ 28	504 (93.5%)	391 (98.2%)	113 (80.1%)	
< 28	35 (6.49%)	7 (1.76%)	28 (19.9%)	
Gender				0.255
Male	284 (52.7%)	216 (54.3%)	68 (48.2%)	
Female	255 (47.3%)	182 (45.7%)	73 (51.8%)	
BW (grams)				< 0.001
≥ 1000	489 (90.7%)	390 (98.0%)	99 (70.2%)	
< 1000	50 (9.28%)	8 (2.01%)	42 (29.8%)	
One-minute Apgar scores	7.50 ± 1.78	7.67 ± 1.72	7.01 ± 1.87	< 0.001
Five-minute Apgar scores	9.04 ± 1.08	9.11 ± 1.11	8.82 ± 0.97	0.003
Feeding patterns				< 0.001
Formula feeding	170 (31.5%)	152 (38.2%)	18 (12.8%)	
Mixed feeding	357 (66.2%)	235 (59.0%)	122 (86.5%)	
Breast feeding	12 (2.23%)	11 (2.76%)	1 (0.71%)	
Respiratory support type				< 0.001
Invasive ventilation	210 (39.0%)	124 (31.2%)	86 (61.0%)	
Noninvasive ventilation	250 (46.4%)	197 (49.5%)	53 (37.6%)	
Others	79 (14.7%)	77 (19.3%)	2 (1.42%)	
Antenatal glucocorticoids (times)				0.038
0	249 (46.2%)	196 (49.2%)	53 (37.6%)	
1	34 (6.31%)	27 (6.78%)	7 (4.96%)	
2	68 (12.6%)	51 (12.8%)	17 (12.1%)	
3	19 (3.53%)	14 (3.52%)	5 (3.55%)	
4	169 (31.4%)	110 (27.6%)	59 (41.8%)	
UVC				< 0.001
No	316 (58.6%)	278 (69.8%)	38 (27.0%)	
Yes	223 (41.4%)	120 (30.2%)	103 (73.0%)	
NRDS				< 0.001
No	99 (18.4%)	93 (23.4%)	6 (4.26%)	
Yes	440 (81.6%)	305 (76.6%)	135 (95.7%)	
NEC				0.009
No	452 (83.9%)	344 (86.4%)	108 (76.6%)	
Yes	87 (16.1%)	54 (13.6%)	33 (23.4%)	
IVH				0.073
No	156 (28.9%)	124 (31.2%)	32 (22.7%)	
Yes	383 (71.1%)	274 (68.8%)	109 (77.3%)	
Pneumorrhagia				< 0.001
No	463 (85.9%)	359 (90.2%)	104 (73.8%)	
Yes	76 (14.1%)	39 (9.80%)	37 (26.2%)	
Sepsis				< 0.001
No	331 (61.4%)	283 (71.1%)	48 (34.0%)	
Yes	208 (38.6%)	115 (28.9%)	93 (66.0%)	
CHD				0.147
PDA	187 (34.7%)	135 (33.9%)	52 (36.9%)	
ASD	55 (10.2%)	34 (8.54%)	21 (14.9%)	
Others	4 (0.74%)	4 (1.01%)	0 (0.00%)	
VSD	11 (2.04%)	9 (2.26%)	2 (1.42%)	
No	282 (52.3%)	216 (54.3%)	66 (46.8%)	
Amniotic fluid contamination				0.240
No	496 (92.0%)	370 (93.0%)	126 (89.4%)	
Yes	43 (7.98%)	28 (7.04%)	15 (10.6%)	
Placental abruption				0.311
No	491 (91.1%)	366 (92.0%)	125 (88.7%)	
Yes	48 (8.91%)	32 (8.04%)	16 (11.3%)	
Hemolytic jaundice				0.018
No	497 (92.2%)	360 (90.5%)	137 (97.2%)	
Yes	42 (7.79%)	38 (9.55%)	4 (2.84%)	
Rescue frequency (≥ 3 times)				< 0.001
No	498 (92.4%)	382 (96.0%)	116 (82.3%)	
Yes	41 (7.61%)	16 (4.02%)	25 (17.7%)	
Maternal age	31.7 ± 4.37	31.5 ± 4.33	32.4 ± 4.40	0.033
Primipare:				0.036
No	218 (40.4%)	172 (43.2%)	46 (32.6%)	
Yes	321 (59.6%)	226 (56.8%)	95 (67.4%)	
Multiple births				0.016
No	424 (78.7%)	324 (81.4%)	100 (70.9%)	
Triplets	1 (0.19%)	1 (0.25%)	0 (0.00%)	
Twins	114 (21.2%)	73 (18.3%)	41 (29.1%)	
Cesarean section				0.997
No	109 (20.2%)	81 (20.4%)	28 (19.9%)	
Yes	430 (79.8%)	317 (79.6%)	113 (80.1%)	
Pregnancy anemia				1.000
No	526 (97.6%)	388 (97.5%)	138 (97.9%)	
Yes	13 (2.41%)	10 (2.51%)	3 (2.13%)	
HDP				0.008
No	342 (63.5%)	266 (66.8%)	76 (53.9%)	
Yes	197 (36.5%)	132 (33.2%)	65 (46.1%)	
GDM				0.293
No	413 (76.6%)	310 (77.9%)	103 (73.0%)	
Yes	126 (23.4%)	88 (22.1%)	38 (27.0%)	
ICP				0.374
No	525 (97.4%)	389 (97.7%)	136 (96.5%)	
Yes	14 (2.60%)	9 (2.26%)	5 (3.55%)	
Hypothyroidism				0.491
No	466 (86.5%)	347 (87.2%)	119 (84.4%)	
Yes	73 (13.5%)	51 (12.8%)	22 (15.6%)	
ITP				0.736
No	528 (98.0%)	389 (97.7%)	139 (98.6%)	
Yes	11 (2.04%)	9 (2.26%)	2 (1.42%)	
WBC	11.5 ± 7.42	12.0 ± 7.02	10.1 ± 8.32	0.017
Platelet	219 ± 78.3	221 ± 79.7	214 ± 74.0	0.367
RDW	16.8 ± 1.95	16.8 ± 1.95	17.0 ± 1.94	0.289
Hct	48.3 ± 9.88	47.8 ± 10.4	49.5 ± 8.26	0.050
Neutrophils	7.74 ± 6.07	8.14 ± 5.85	6.60 ± 6.56	0.015
Lymphcytes	2.37 ± 1.27	2.45 ± 1.29	2.14 ± 1.19	0.008
PCT	10.8 ± 19.0	10.6 ± 18.7	11.4 ± 20.0	0.666
CRP	14.0 ± 14.3	14.2 ± 15.1	13.4 ± 11.6	0.495
IL	225 ± 651	210 ± 614	265 ± 746	0.432
K	4.20 ± 0.90	4.14 ± 0.86	4.38 ± 0.98	0.012
Na	138 ± 5.10	137 ± 5.08	139 ± 5.05	0.011
PH	7.33 ± 0.10	7.32 ± 0.09	7.35 ± 0.10	0.002
PO_2_	99.8 ± 43.3	97.0 ± 42.0	108 ± 46.1	0.018
PCO_2_	36.9 ± 12.0	37.7 ± 12.0	34.7 ± 11.8	0.011
HCO_3_	19.2 ± 3.56	19.5 ± 3.61	18.3 ± 3.25	< 0.001
TBIL	120 ± 49.6	120 ± 51.4	117 ± 44.1	0.486
ALB	28.0 ± 3.87	28.4 ± 3.92	26.9 ± 3.53	< 0.001
AST	63.4 ± 50.2	66.1 ± 53.0	55.8 ± 40.5	0.017
ALT	16.4 ± 26.8	17.7 ± 30.7	12.9 ± 8.28	0.005
LDH	744 ± 545	748 ± 587	732 ± 407	0.719
CK	277 ± 394	303 ± 439	202 ± 207	< 0.001
LA	5.63 ± 2.29	5.63 ± 2.26	5.60 ± 2.38	0.891
TG	0.82 ± 0.61	0.80 ± 0.60	0.86 ± 0.63	0.390
TC	2.33 ± 0.79	2.30 ± 0.79	2.40 ± 0.79	0.204
LDL	1.25 ± 0.58	1.24 ± 0.57	1.28 ± 0.61	0.464
HDL	0.76 ± 0.32	0.74 ± 0.32	0.80 ± 0.29	0.048
Creatinine	69.9 ± 20.9	69.0 ± 20.8	72.4 ± 21.0	0.102
Urea	5.85 ± 2.74	5.58 ± 2.52	6.64 ± 3.15	< 0.001

*GA* gestational age, *BW* birth weight, *UVC* umbilical venous catheterization, *NRDS* neonatal respiratory distress syndrome, *NEC* necrotizing enterocolitis, *IVH* intraventricular hemorrhage, *CHD* congenital heart disease, *PDA* patent ductus arteriosus, *ASD* atrial septal defect, *VSD* ventricular septal defect, *HDP* hypertensive disorders of pregnancy, *GDM* gestational diabetes mellitus, *ICP* intrahepatic cholestasis of pregnancy, *ITP* idiopathic thrombocytopenic purpura, *WBC* white blood cell, *PLT* platelet, *RDW* red cell distribution width, *HCT* hematocrit, *PCT* procalcitonin, CRP C-reactive protein, *IL* interleukin, K potassium, Na sodium, *PH* potential of hydrogen, PaO_2_ arterial partial pressure of oxygen, PaCO_2_ arterial partial pressure of carbon dioxide, HCO_3_ bicarbonate, *TBIL* total bilirubin, *ALB* albumin, *AST* aspartate aminotransferase, *ALT* alanine aminotransferase, *LDH* lactate dehydrogenase, *CK* creatine kinase, *LA* lactic acid, *TG* triglyceride, *TC* total cholesterol, *LDL* low-density lipoprotein, *HDL* high-density lipoprotein.

### Variables selection

**Figure 2:**
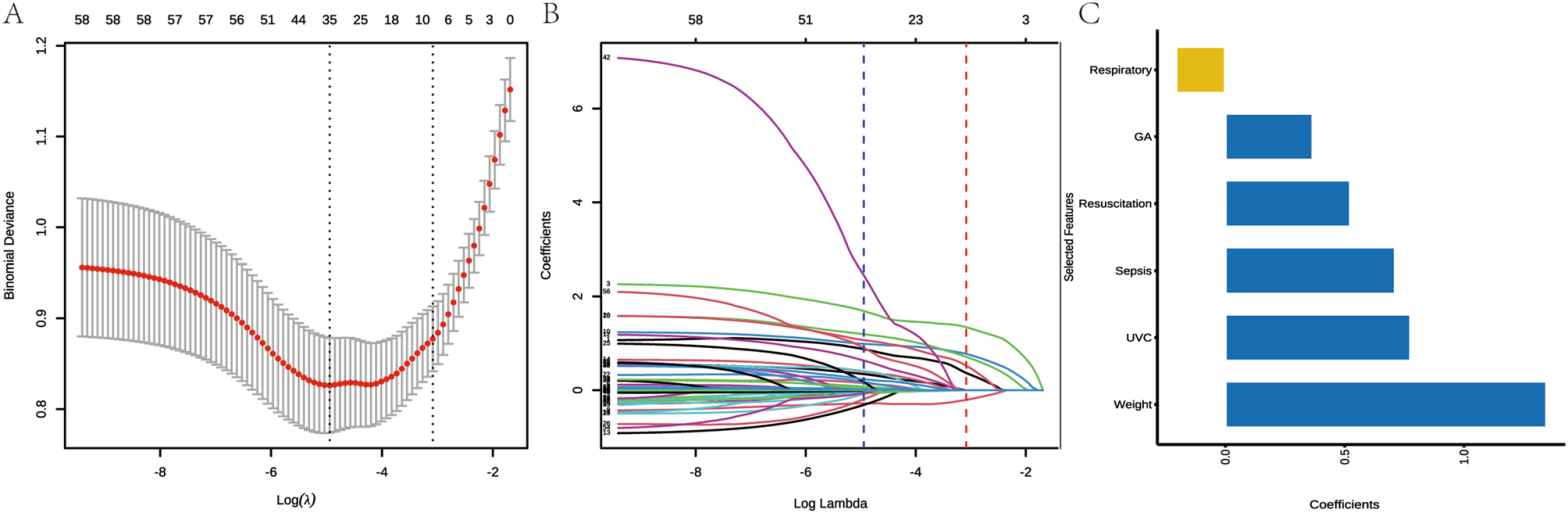
Feature selection. (**A**) Variable selection using LASSO logistic regression model. The dashed line on the left represents the minimum criterion, and the 1-SE of the minimum criterion is used to determine the optimal parameter (lambda) selection in the LASSO model (represented by the dashed line on the right). (**B**) Silhouette of LASSO coefficients for 57 features. (**C**) Features with non-zero coefficients selected by LASSO.;

Based on the data from the training set, we conducted LASSO regression analysis to identify independent predictor variables that significantly affect long LOS. The LASSO analysis yielded a reduction of the initial 57 perinatal variables down to six potential predictors, resulting in a ratio of 9.5:1 (Fig. 2A,B). The six potential predictors identified through the LASSO analysis were GA (< 28 weeks), BW(< 1000 g), respiratory support type, umbilical venous catheter (UVC) use, sepsis, and rescue frequency (≥ 3 times) (Fig. 2C).

### Risk prediction nomogram development

**Figure 3:**
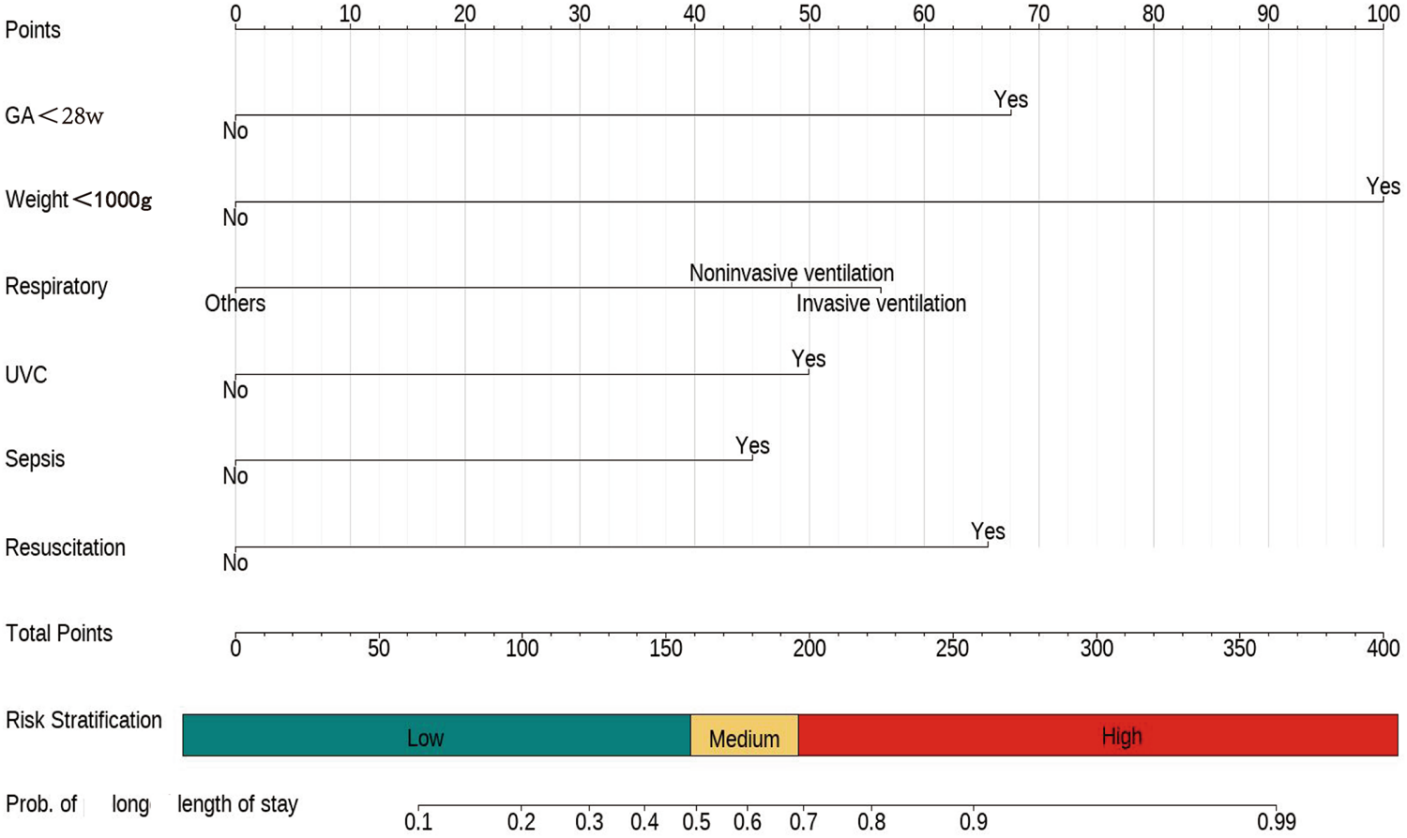
A nomogram predicting the risk of long Length of stay in infant with blood transfusions. The long LOS risk nomogram was developed in the cohort, with GA, BW, Respiratory support type, UVC, sepsis and rescue frequency.

A logistic regression model was constructed using the six predictor variables identified by LASSO: GA (< 28 weeks), BW(< 1000 g), respiratory support type UVC use, sepsis, and rescue frequency (≥ 3 times). Table 2 presents the logistic regression model, displaying the coefficients and corresponding *p*-values for each of the six predictor variables. These coefficients indicate the strength and direction of the association between the predictor variables and the outcome (long LOS). As shown in Fig. 3, Each blood transfusion patient’s risk of an extended hospital stay for can be estimated by evaluating the cumulative points assigned on the nomogram. A higher total score indicates a greater likelihood of long hospitalization. Previous research has identified specific clinical features as predictor variables for long LOS. To enhance our model, we incorporated additional features and evaluated their discriminatory ability (Table 3). However, the results indicated that adding these predictor variables to the validation set did not lead to significant improvements, and may have led to models that were overfitted. Consequently, we have decided to use the nomogram as our final model.

**Table 2:** Predictive factors for long LOS in infants undergoing blood transfusion.

Characteristic	OR^1^	95% CI^1^	*p*-value
GA (weeks)			
≥ 28	–	–	
< 28	2.79	1.01, 8.17	0.051
BW (grams)			
≥ 1000	–	–	
< 1000	7.22	3.01, 19.2	< 0.001
Respiratory support			
Invasive ventilation	–	–	
Noninvasive ventilation	0.70	0.43, 1.16	0.2
Others	0.20	0.03, 0.71	0.033
UVC			
No	–	–	
Yes	3.15	1.91, 5.25	< 0.001
Sepsis			
No	–	–	
Yes	3.23	2.00, 5.27	< 0.001
Rescue frequency (≥ 3 times):			
No	–	–	
Yes	3.87	1.74, 8.82	0.001

*GA* gestational age, *BW* birth weight, *UVC* umbilical venous catheterization, *OR*^*1*^ Odds Ratio, *CI*^*1*^ Confidence Interval.

**Table 3:** Different model performance.

Models	Auc	Sen	Spe	Acc	Pre	Npv	Ppv	Rec	*p*
Model 1 (M1a)	0.842	0.424	0.972	0.831	0.84	0.83	0.84	0.424	
Model 2 (M2a)	0.858	0.505	0.941	0.829	0.746	0.846	0.746	0.505	
Model 3 (M3a)	0.86	0.525	0.93	0.826	0.722	0.85	0.722	0.525	
M1a versus M2a									0.058
M1a versus M3a									0.074
M2a versus M3a									0.744
Model 1 (M1b)	0.851	0.5	0.938	0.818	0.75	0.833	0.75	0.5	
Model 2 (M2b)	0.849	0.524	0.938	0.825	0.759	0.84	0.759	0.524	
Model 3 (M3b)	0.839	0.524	0.911	0.805	0.688	0.836	0.688	0.524	
M1b versus M2b									0.837
M1b versus M3b									0.427
M2b versus M3b									0.41

*Auc* area under the curve, *Sen* sensitivity, *Spe* specificity, *Acc* accuracy, *Pre* precision, *Ppv* positive predictive value, *Npv* negative predictive value.

M1a, M2a, and M3a represent the apparent performance of the models fitted using the training set, while M1b, M2b, and M3b correspond to the performance of the models evaluated using the validation set. *P means the Delong test that compare the AUC value of different models.

M1 represents the final model illustrated in the nomogram.M2 introduces RDS, NEC, and pneumorrhagia; M3 introduces RDS, NEC, pneumorrhagia, 1-min and 5-min Apgar scores, HDP, and ALB.

### Evaluation of the performance of the predictive model

**Figure 4:**
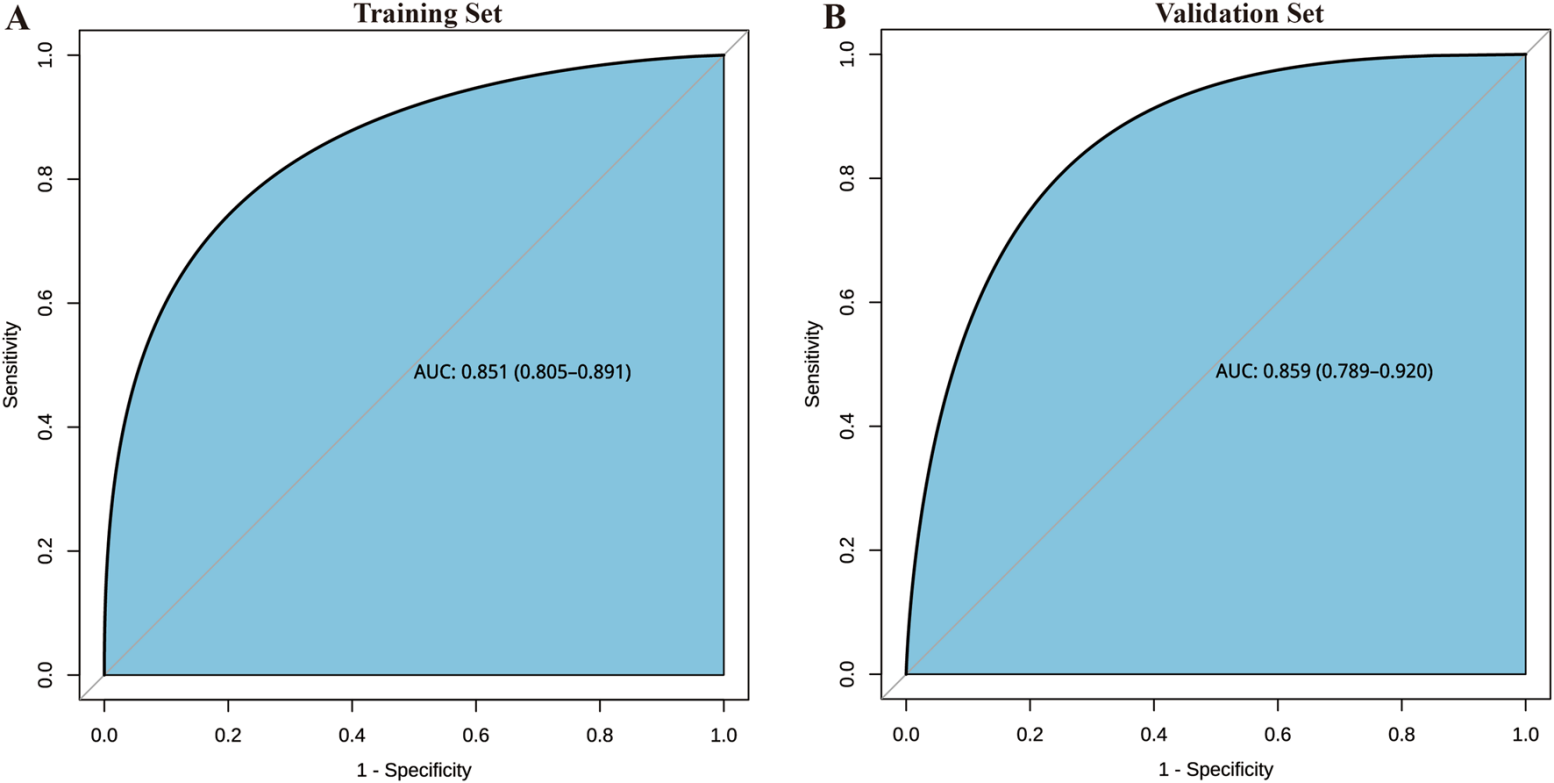
ROC curves. (**A**) Training set (ROC). (**B**) Validation set (ROC). ROC = receiver operating characteristic, AUC = area under the ROC curve.

**Figure 5:**
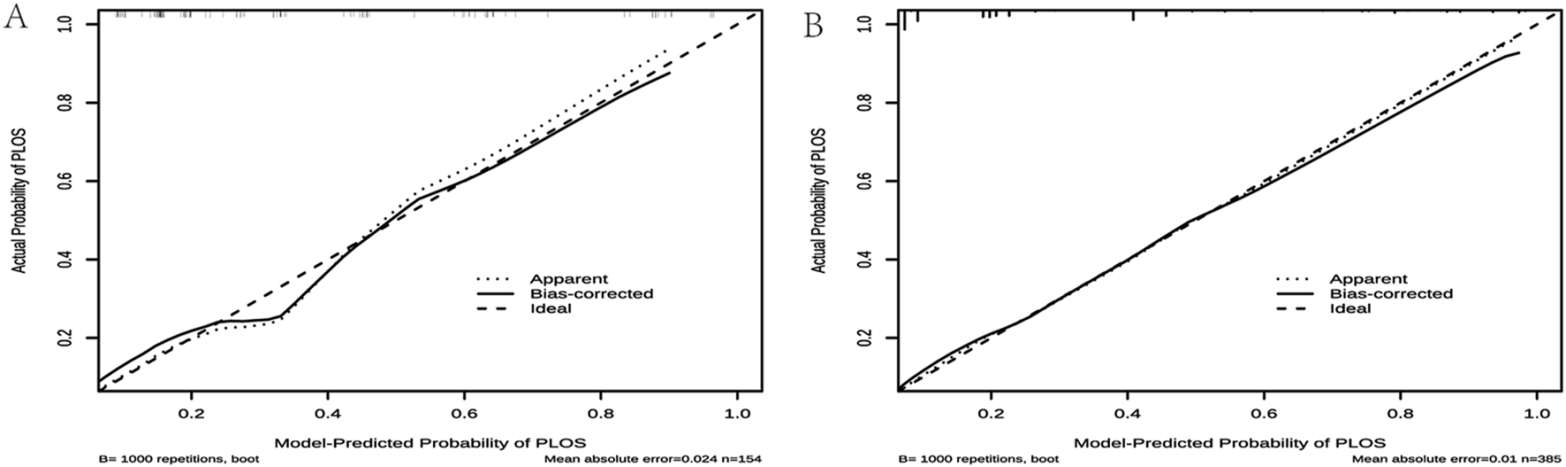
Calibration curves. (**A**) Assessing agreement between predicted probabilities of long LOS and observed outcomes within the training set. (**B**) Assessing agreement between predicted probabilities of long LOS and observed outcomes within the validation set. X-axis: Predicted probabilities; Y-axis: Observed proportions of long LOS. Deviations from the ‘Ideal’ line indicate potential errors. Points aligning with the ‘Ideal’ line indicate good calibration. The ‘Apparent’ estimate is uncorrected and biased. ‘Bias-corrected’ estimate improves accuracy. ‘Ideal’ estimate serves as a benchmark. Tick marks show percentiles. Mean Absolute Error (MAE) measures overall accuracy. Sample size (n) reported for each estimate.

**Figure 6:**
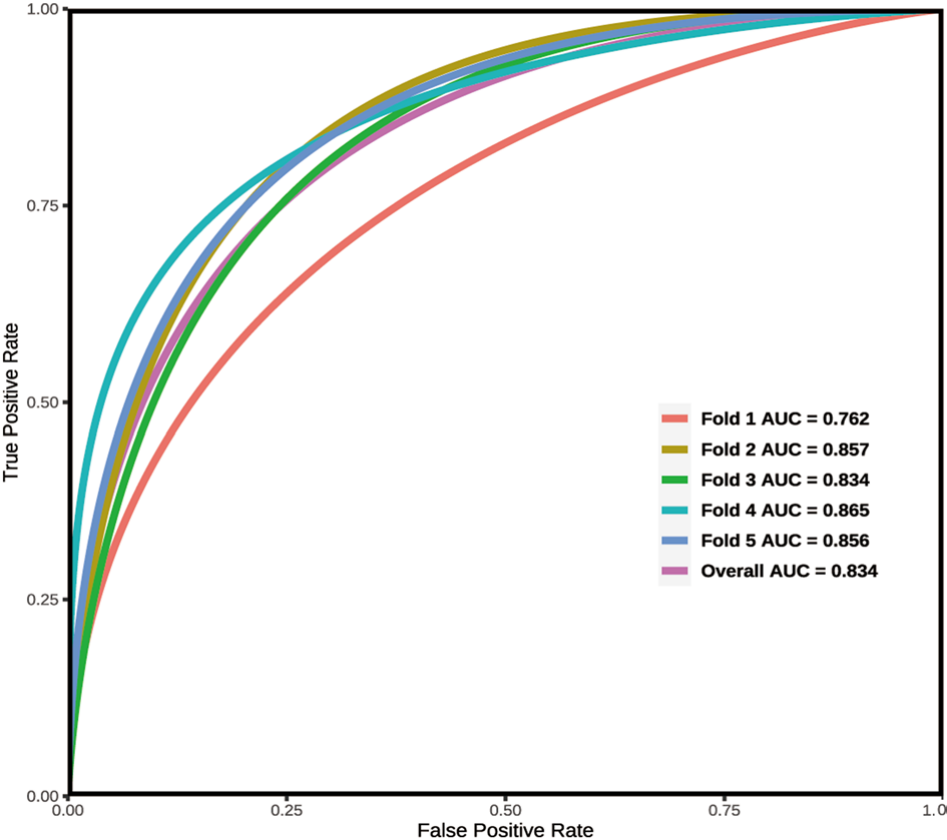
Fivefold cross-validation. Fivefold cross-validation involves dividing the dataset into 5 equal parts, where 4 parts are used for training and 1 part is used for validation. This process is repeated 5 times, with each different subset serving as the validation set. The results are then averaged to provide an evaluation metric for the model’s performance.

The differentiation capacity of the developed model was validated in both the training set and validation set. The AUC for the nomogram in the training set was 0.851 (95% CI 0.805–0.891), as shown in Fig. 4A. Similarly, in the validation set, the AUC was 0.859 (95% CI 0.789–0.920), as depicted in Fig. 4B. These results indicate that the model exhibited good discriminatory and predictive abilities. The calibration curve demonstrated that the model exhibited an excellent ability to accurately predict actual probabilities, as shown in Fig. 5A,B. Based on 1000 rounds of resampling, the mean absolute error (MAE) obtained for the training set calibration curve was 0.024 with a sample size of 154. Similarly, for the validation set calibration curve, the MAE achieved through 1000 rounds of resampling was 0.01 with a sample size of 385.The *Hosmer–Lemeshow* test indicated no significant difference between our model and the observed values (*p* > 0.05). The R2 of our model was 0.331. Furthermore, we conducted an assessment using fivefold cross-validation to evaluate the generalizability of our model. The results, depicted in Fig. 6, demonstrate satisfactory performance.

### Clinical effectiveness of the model

**Figure 7:**
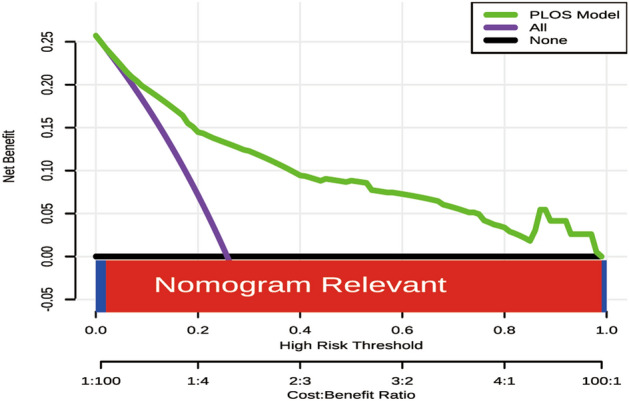
Decision curve analysis of nomograms. Using the nomogram to predict long length of stay is the optimal decision-making strategy for maximizing net benefit, especially when compared to scenarios where no prediction model is utilized (i.e., treat-all or treat-none scheme) across a majority of given threshold probabilities (> 2%).

Figure 7 displays the decision curve analysis for the long LOS nomogram for children undergoing blood transfusion. This analysis reveals that the model is relevant across a wide range of risk thresholds.

## Discussion

The LOS in the NICU has been a focal point of research. There exists a correlation between long LOS and hospital-acquired conditions, as well as adverse events in healthcare^16,17^. Blood transfusion is a frequently performed procedure for neonates who require intense care, especially for preterm neonates. However, there is limited literature available on predicting LOS specifically for this high-risk patient population.

Early identification of long LOS-NICU risk in NICU neonatal patients who received transfusion therapy is not only crucial for providing important counseling references to families but may also guide decisions on optimal clinical interventions. Thus, in this study, we utilized historical clinical data from the NICU to identify important predictor variables and developed a predictive model for long NICU LOS in neonates receiving blood transfusions. We deployed routinely used machine learning algorithm (LASSO) to selection the predictor variables related to aforementioned issue. We identified six independent characteristics in our study: GA (< 28 weeks), BW(< 1000 g), Respiratory support type, UVC, sepsis and rescue frequency (≥ 3 times). Our model demonstrated excellent predictive performance in both the training and validation set, with discrimination abilities of 0.851 (95% CI 0.805–0.891) and 0.859 (95% CI 0.789–0.920) respectively. These results indicate strong predictive power and suggest excellent performance in accurately identifying long LOS.

The GA and BW are frequently used to evaluate newborn infants. We found that GA lower than 28 weeks and BW less than 1000 g dramatically increased the probability of long LOS. Our findings are consistent with previous research indicating that birth weight and gestational age are the primary predictor variables influencing long length of stay in the NICU^18,19^. The incidence of anemia is high among premature infants. This is due to several factors, including their smaller circulating blood volume, shorter lifespan of red blood cells, and an immature bone marrow response to anemia. The immature hepatic receptors in premature infants are relatively insensitive to tissue hypoxia, and their plasma erythropoietin (EPO) levels are also low^20^. The degree of deficiency is especially significant in the smallest and least mature infants^21^. Another crucial factor is that premature infants require careful monitoring of various parameters, which often involves repeated blood sampling for laboratory analysis. During the initial hospitalization period, premature infants may require a greater number of red blood cell transfusions compared to full-term infants in order to elevate hemoglobin levels and improve blood oxygenation capacity^22^.

In this study, blood-transfused NICU who required respiratory support such as mechanical ventilation during hospitalization generally had longer hospital stays compared to those who only needed supplemental oxygen or do not require respiratory support. This finding is similar to previous research^23^. This means that there is a strong and consistent correlation between the need for invasive respiratory support and the length of hospital stay in transfusion-dependent infants. This may be attributed to the fact that patients who require invasive respiratory support may have more severe conditions, such as serious illnesses or injuries, that necessitate ongoing supportive treatments including blood transfusions. Transfusions can help improve oxygenation levels by providing sufficient hemoglobin and oxygen transport^23,24^. On the other hand, they may face a higher risk of complications such as infections^25^, which can further prolong hospitalization. Moreover, patients requiring mechanical ventilation may take longer to wean off the ventilator and may require respiratory physiotherapy after extubating to promote lung function recovery. Therefore, the need for invasive respiratory support is an important predictor variable in predicting the length of hospital stay in transfusion-dependent patients. Healthcare professionals should consider this when developing patient care plans and allocating hospital resources. Appropriate management of invasive respiratory support may help shorten the length of hospital stay and potentially improve patient prognosis.

UVC is a common invasive procedure often used in neonates^26^. This procedure involves inserting a catheter into the neonate’s umbilical vein for purposes such as blood transfusion, fluid administration, medication delivery, or monitoring hemodynamics^27^. While umbilical vein catheterization is considered effective and safe, it is important to note that the UVC is associated with longer duration of hospitalization. The procedure involves local anesthesia, manipulation, and fixation of neonates, which may cause discomfort or complications such as infection and bleeding^28–30^. As a result, hospitalization time for neonates requiring blood transfusion may be extended. On the other hand, in some cases of severely ill neonates, umbilical vein catheterization may be necessary for ongoing treatment or monitoring. In such situations, the presence of the umbilical vein catheter extends the duration of hospitalization for the child, requiring longer periods of observation and treatment for transfusion-dependent neonates.

The occurrence of nosocomial infection in hospitalized newborns is prevalent and carries significant consequences. It is widely recognized as one of the most frequently encountered adverse events during their hospitalization^31^. The immune system of newborns is relatively weak, with poor resistance, making them vulnerable to various pathogens. Infections can lead to severe complications and even endanger lives^32,33^. In our study, predictor variables contributing to long LOS in transfused children included sepsis. Previous studies have also confirmed that sepsis is a risk factor for long hospitalization^10,34,35^. Sepsis may require long-term symptom management, antimicrobial therapy, shock management, nutritional support, and carry a high risk of complications, thereby extending treatment time and LOS. Research has shown that the median LOS for infected newborns is twice that of uninfected newborns^19^. This indicates that infections do have a significant impact on the length of hospital stay for newborns. The occurrence of sepsis in transfused children may necessitate additional treatment and rehabilitation processes, also increasing medical costs and family burden. In our study, we have identified an important predictor variable affecting the length of hospital stay for newborns, which is the number of resuscitation attempts. This finding, which has not been previously mentioned in existing research, may be attributed to variations in healthcare policies across different regions. Newborns requiring multiple resuscitation attempts, particularly those in need of blood transfusions, often have severe conditions with multiple organ dysfunctions or systemic diseases. These infants require long medical monitoring, treatment, and rehabilitation to stabilize their condition. Additionally, frequent resuscitation attempts can induce fatigue and physical stress in newborns. To ensure their safety and stability, healthcare teams typically extend the duration of hospital observation, providing necessary rehabilitation and monitoring to prevent further deterioration.

Our research provides healthcare professionals with a visual predictive tool for identifying transfused infants at higher risk of long LOS. This allows clinicians to differentiate infants with a higher risk of long LOS, enabling them to plan general resource allocation accordingly. By identifying high-risk patients in advance, clinicians can plan for sufficient beds, equipment, and staff in the neonatal intensive care unit. The LOS prediction model also identifies potentially modifiable predictor variables that are associated with long hospital stays in transfused infants. The impact of modifying care to optimize these predictor variables could be studied in future research.

## Limitations

The present study has several limitations that should be acknowledged. First, the discharge standards across hospitals may differ, which may confound the results. Second, although our model’s internal validation demonstrated excellent calibration and discrimination, external validation is still required using additional datasets to confirm its reliability. Third, as our study was conducted at the largest children’s medical center in the region, there may be a selection bias towards critically ill premature infants. Thus, generalizing our findings to other healthcare settings with different patient populations should be done with caution. Furthermore, to establish the robustness and reliability of our results, prospective studies conducted in multicenter clinical trials are needed. Finally, future research could explore including additional predictive variables to improve the model’s performance and predictive capabilities.

## Conclusions

The present study introduced a novel nomogram that demonstrates satisfactory accuracy in assisting clinicians to assess the risk of long LOS in infants undergoing blood transfusion. GA (< 28 weeks), BW(< 1000 g), respiratory support type, UVC, sepsis and rescue frequency (≥ 3 times) were related to an increased risk of long length of NICU stay in infants receiving blood transfusion. This model can help healthcare professionals stratify the risk level of long hospital stay for children undergoing blood transfusion in NICU, conduct appropriate clinical interventions, and effectively allocate medical resources.

## Data Availability

Upon reasonable request, the corresponding authors of this article will provide unrestricted access to the original data.
